# Filling Vacant Chairs by Private Application

**Published:** 1847-06

**Authors:** 


### ARTICLE VI.

### FILLING VACANT CHAIRS BY PRIVATE APPLICATIONS.

This plan, adopted by Transylvania University last year to fill the vacant chair of Obstetrics in the medical department of that school is the nearest approach, in this country, to the system of concours, which we ardently hope yet to see adopted by all colleges. In the instance above referred to the system of private applications seems to have worked well.

The University of the city of New York, we observe by an advertisement, has determined upon the same plan to fill the vacant chair of Theory and Practice in that school. By it "The Medical Profession are informed that applications for this professorship will be received until August 10th." Communications to be addressed to John W. Draper, M. D., Secretary of the Faculty, No. 364, Fourth Street, New York. The name of no applicant except the successful one, to be made known.

This we regard as tending to the system of public trials.

It has some advantages, but we think great disadvantages. It is a good test of an individual's standing in community by showing the number and character of the recommendations he can muster. But all know that many good men and talented practitioners, with great influence in society, are yet wholly unqualified for public teaching. While it gives all an opportunity to "try," there cannot be that confidence in its impartiality there would be if the trial were a public one. Here, too, the applicant, unless successful, gains nothing; when, if public, he would have the additional inducement of a prospect of distinguishing himself and of thus being placed in a favorable light for a succeeding contest.

